# Laparoscopic liver resection after laparoscopic pancreatoduodenectomy for liver metastases of ampulla of Vater adenocarcinoma

**DOI:** 10.1186/s40792-020-01043-0

**Published:** 2020-10-08

**Authors:** Muneyasu Kiriyama, Yuji Kaneoka, Atsuyuki Maeda, Yuichi Takayama, Takamasa Takahashi, Kazuaki Seita

**Affiliations:** Department of Surgery, Ogaki Municipal Hospital, 4-86, Minaminokawa, Ogaki, Gifu 503-0864 Japan

**Keywords:** Laparoscopic liver resection, Laparoscopic pancreatoduodenectomy, Ampulla of vater adenocarcinoma, Liver metastasis

## Abstract

**Background:**

Repeat laparoscopic surgery has become increasingly common. However, reports of liver resection after pancreatoduodenectomy are scarce, and we report the first successful case of a patient who underwent laparoscopic liver resection after laparoscopic pancreatoduodenectomy.

**Case presentation:**

A 65-year-old man underwent laparoscopic pancreatoduodenectomy for ampulla of Vater adenocarcinoma. According to the American Joint Committee on Cancer (8th edition) staging guidelines, the tumour was labelled as stage IIIB (fT2N2M0). Twelve months later, a computed tomography (CT) scan revealed liver masses (in segments 3 and 5) and swollen para-aortic lymph nodes. After six chemotherapy courses of gemcitabine with cisplatin, the CT scan showed the disappearance of the para-aortic lymph nodes and progression of liver metastases. Nineteen months after the initial surgery, the patient underwent laparoscopic partial liver resection of segment 5 and left lateral sectionectomy. First, we performed the operation in the left half lateral decubitus position. In this position, the portal vein was isolated safely without hindering the hepato-jejunal anastomosis, although the adhesions around the hepato-jejunal anastomosis were dense. Therefore, we were able to perform liver transection safely with vascular inflow control. The operation duration was 235 min, and the volume of blood loss was 100 g. Macroscopically, the resected margins were negative. The patient was uneventfully discharged 12 days after the second operation. Afterwards, drainage was needed because of an intra-abdominal abscess. Currently, he has been alive for 8 months postoperatively, receives chemotherapy to suppress para-aortic lymph node metastases, and has not had another recurrence.

**Conclusions:**

Liver resection after pancreatoduodenectomy can be performed safely with an innovative body position to isolate the portal vein, which is a key point of the surgery. A laparoscopic approach for liver resection after pancreatoduodenectomy is a feasible option.

## Background

The laparoscopic approach has been used in cases of repeat surgery [1, 2]. However, for liver resection (LR) after pancreatoduodenectomy (PD), achieving vascular inflow control, such as with the Pringle manoeuvre or liver parenchymal transection, is often difficult because of dense adhesions [3, 4]. Here, we report the first successful case of a patient who underwent laparoscopic liver resection (LLR) after laparoscopic pancreatoduodenectomy (LPD).

## Case presentation

**Fig. 1:**
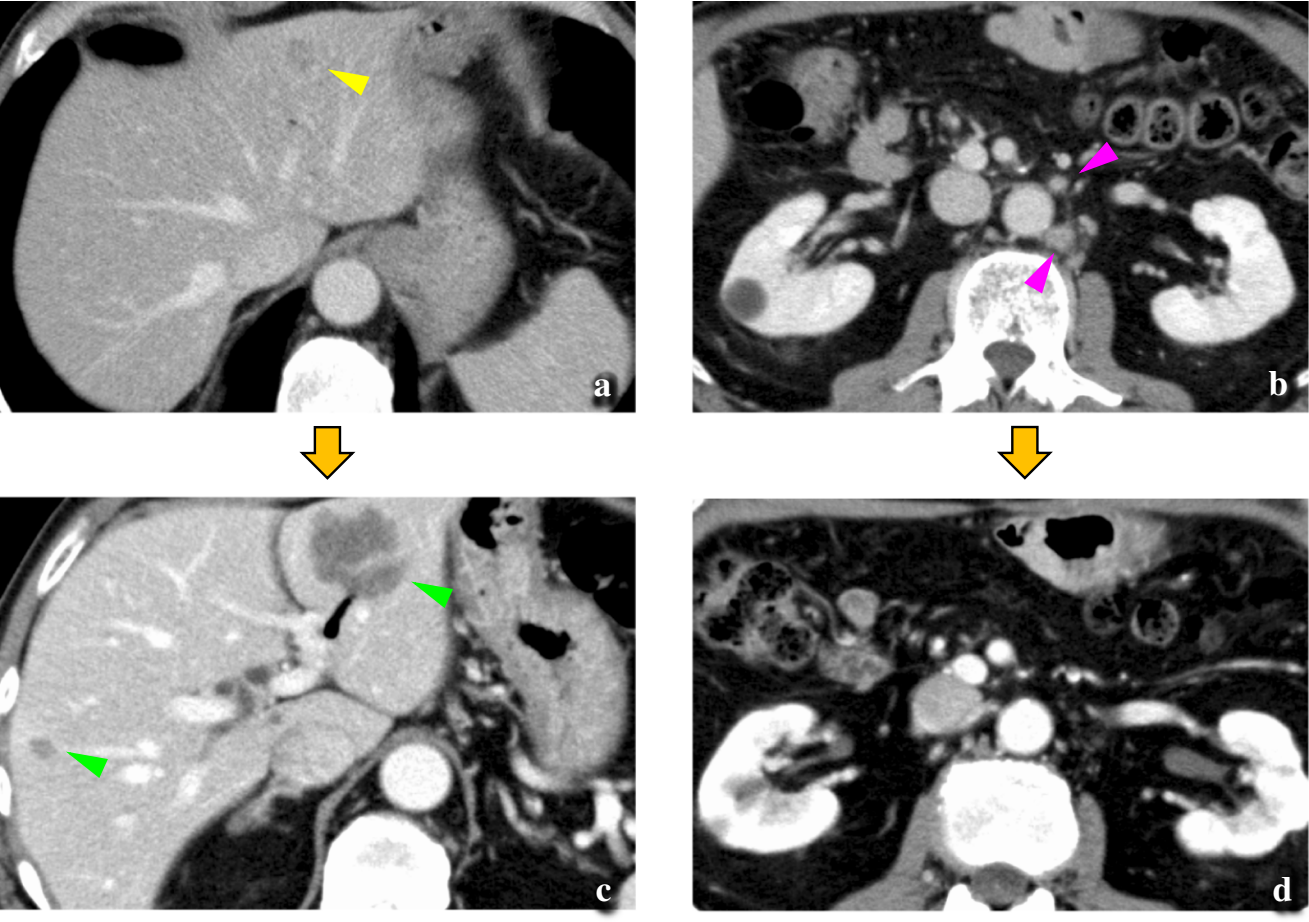
Computed tomography. Liver metastasis in segment 3 (**a** yellow arrowhead) and swollen para-aortic lymph nodes were identified (**b** pink arrowheads) 12 months after laparoscopic pancreatoduodenectomy. After six courses of chemotherapy, the liver deposits progressed in segments 3 and 5 (**c** green arrowheads), while the lymph nodes regressed (**d**)

**Fig. 2:**
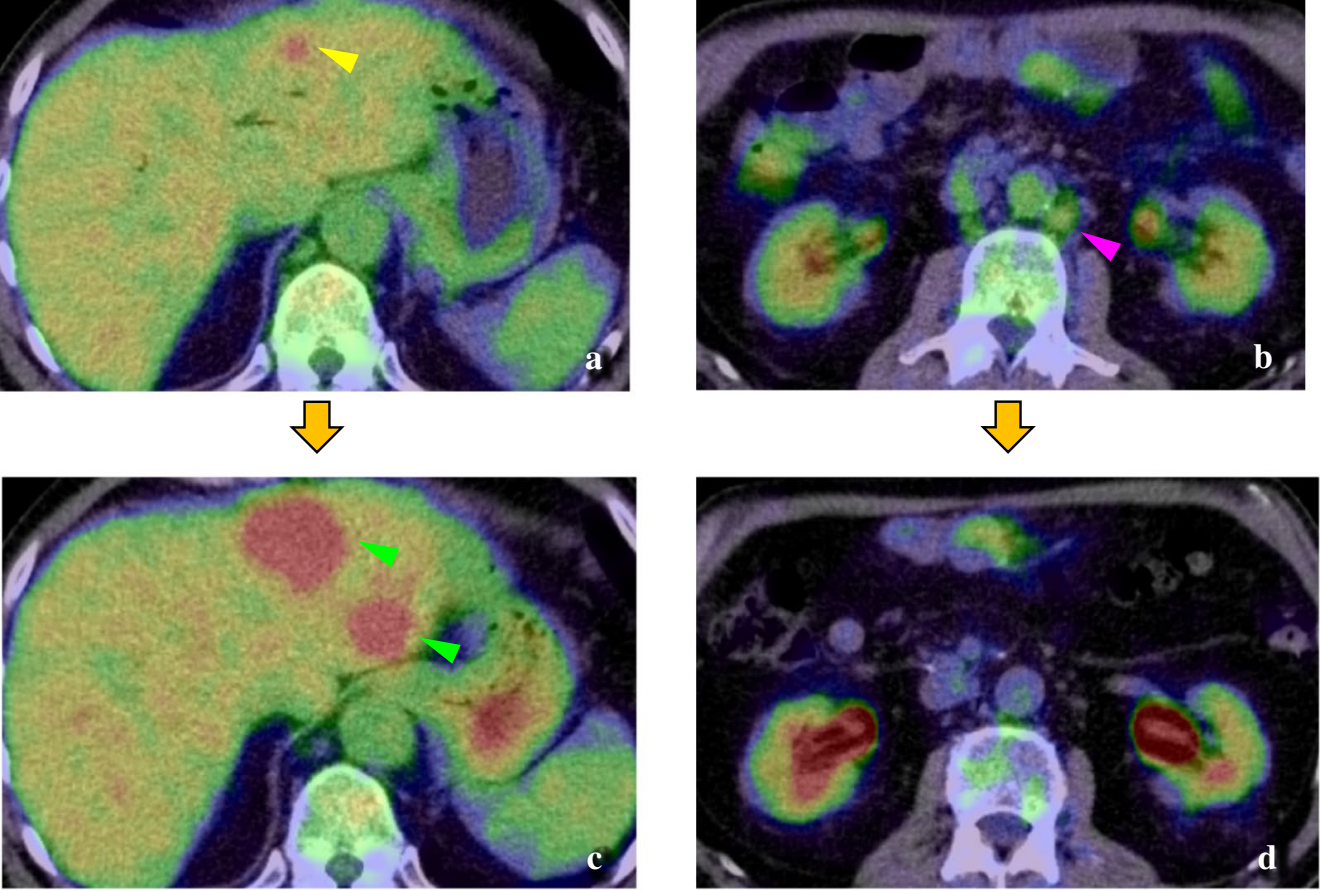
Fluorodeoxyglucose positron emission tomography–computed tomography. The abnormal uptake of the liver metastasis in segment 3 (**a** yellow arrowhead; SUV*_max_ was 4.65) and para-aortic lymph nodes were identified (**b** pink arrowheads; SUV_max_ was 2.53) 12 months after laparoscopic pancreatoduodenectomy. After six courses of chemotherapy, the uptake of the liver lesion was stronger (**c** green arrowheads; SUV_max_ was 7.47), but the uptake of the para-aortic lymph nodes vanished (**d** SUV_max_ was 1.70). *: standardized uptake value

**Fig. 3:**
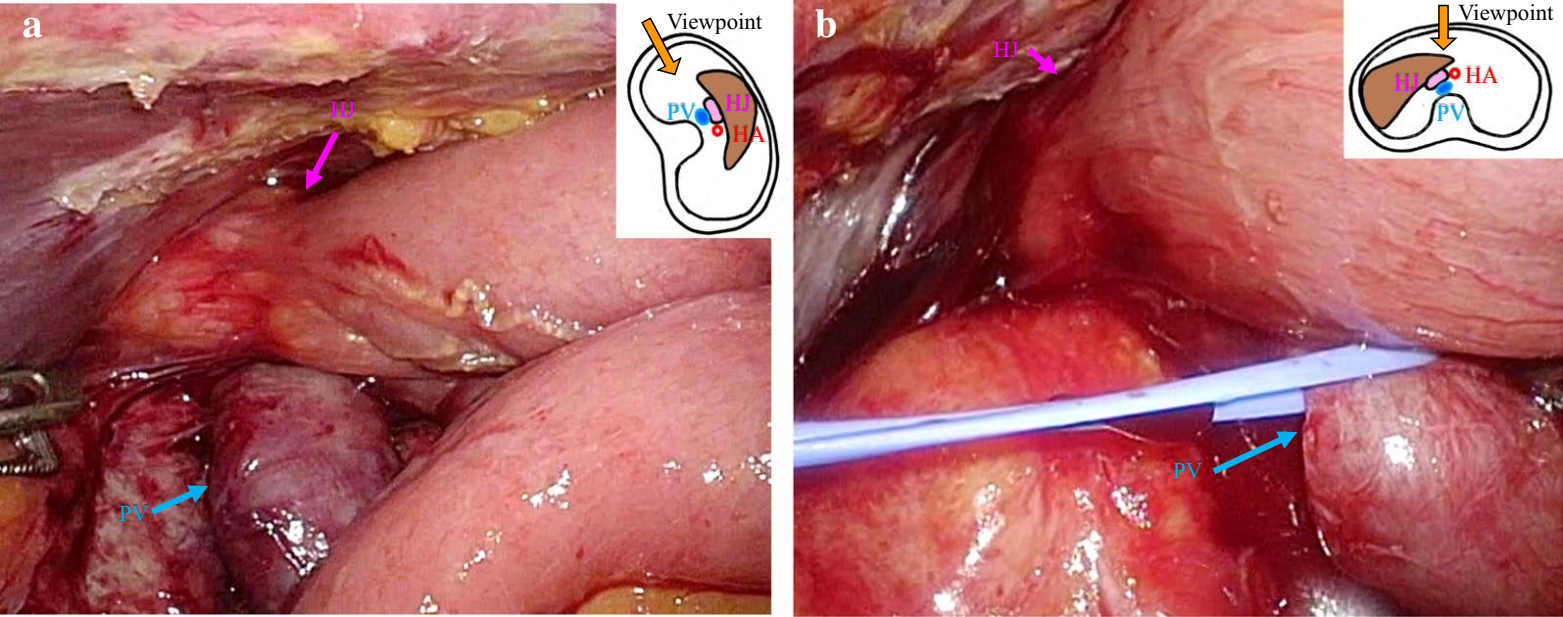
Laparoscopic findings of the hepatic hilum during laparoscopic pancreatoduodenectomy. In the left lateral decubitus position (**a**), the portal vein (PV) was easily encircled beneath the hepaticojejunostomy (HJ). In contrast, in the supine position (**b**), the jejunal loop covered the PV and hindered its identification

A 65-year-old man underwent laparoscopic pylorus-preserving PD with modified child reconstruction for ampulla of Vater adenocarcinoma. We performed standard pylorus-preserving PD for periampullary tumours with six ports. Lymph-node dissection of the caudal half of the hepatoduodenal ligament was performed. Pancreaticojejunostomy and duodenojejunostomy were performed via mini-laparotomy (7 cm), and hepaticojejunostomy was performed laparoscopically. We did not use antiadhesive drugs. In the postoperative course, drainage was needed due to a pancreatic fistula. Macroscopically, papillary tumours spread from the ampulla of Vater to the distal bile duct. Histopathologically, the tumour was moderately differentiated tubular adenocarcinoma that invaded the sphincter of Oddi with four nodal metastases, and all margins were free of tumour cells. According to the American Joint Committee on Cancer (8th edition) staging guidelines, the tumour was labelled as stage IIIB (fT2N2M0) [5]. Twelve months later, a computed tomography (CT) scan revealed liver masses (in segments 3 and 5) and swollen para-aortic lymph nodes (Fig. 1a, b). Fluorodeoxyglucose positron emission tomography (FDG-PET) showed abnormal uptake in both lesions (Fig. 2a, b). He received chemotherapy with gemcitabine plus cisplatin after being diagnosed with liver metastases and para-aortic lymph-node metastases from ampulla of Vater adenocarcinoma. After three courses of chemotherapy, the para-aortic lymph nodes were reduced in size, but the liver metastases did not change on CT. Three courses (six courses in total) later, the CT scan showed the disappearance of the para-aortic lymph nodes and progression of liver metastases (Fig. 1c, d). The uptake of the liver lesion was stronger, but that of para-aortic lymph nodes vanished on FDG-PET (Fig. 2c, d). Moreover, his serum level of the tumour marker carbohydrate antigen 19-9 (CA19-9) increased to 159.9 U/mL. After obtaining his consent, we planned to perform hepatectomy to remove the life-threatening liver metastases (in segments 2, 3, and 5). Nineteen months after the initial surgery, he underwent laparoscopic partial LR of segment 5 and left lateral sectionectomy. First, we performed partial resection of segment 5 with five ports in the half left lateral decubitus position. The intraperitoneal adhesions were somewhat loose, but the adhesions around the hepato-jejunal anastomosis were rather hard. After removing the adhesions from the right dorsal side, we could isolate the portal vein without hindering the hepato-jejunal anastomosis. Furthermore, on the left side of the hepatic duct, the hepatic artery with firm granulation tissue was isolated. Therefore, we could perform partial resection of segment 5 safely by gaining vascular inflow control by occluding the hepatic artery and portal vein with clamp forceps (Fig. 3a). Next, we performed left lateral sectionectomy in the supine position with five ports; three were other ports. However, it was difficult to clamp the portal vein, because it was hidden behind the Roux limb, leading to hepato-jejunal anastomosis (Fig. 3b). The operation duration was 235 min, and the volume of blood loss was 100 g. The histopathologic diagnosis was liver metastases from ampulla of Vater adenocarcinoma with grade 1a histological therapeutic effect, and all surgical margins were negative. The prophylactic drain was removed on the 7th postoperative day. He was uneventfully discharged 12 days after the second operation without fever or abnormal abdominal pain. Afterwards, on the 19th postoperative day, drainage was needed because of an intra-abdominal abscess, which was diagnosed by CT at an emergency room visit. Enteric organisms, including *Citrobacter freundii*, *Klebsiella pneumoniae*, *Enterococcus gallinarum*, and *Streptococcus constellatus*, were identified in the drainage fluid. This additional drain was removed after the abscess healed. Currently, the patient has been alive for 8 months after LLR, and his postoperative CA19-9 levels have decreased to the normal range (36.6 U/mL). Eight months after the second operation, he underwent a chemotherapy regiment that was the same as before the operation, gemcitabine plus cisplatin, to suppress para-aortic lymph-node metastases as planned, and has not had another recurrence.

## Discussion

LR after PD is rare because of the poor prognosis of peripancreatic tumours. A few limited cases have been reported to date [3, 4, 6–8]. During liver surgery after PD, dense adhesions around the hepatic artery and portal vein, which are close to the hepatojejunostomy and pancreaticojejunostomy, make it difficult to isolate these vessels [3, 4]. In particular, the adhesion around the pancreaticojejunostomy after pancreatic fistula on initial operation is expected to be the most severe. Consequently, transection of the liver parenchyma is sometimes needed without vascular inflow control. As approximately 70% of the liver’s blood flow is supplied by the portal system, control of the hepatic artery was considered to be optional. Isolation of the portal vein, which is buried in the most dorsal portion of the dense adhesions, is a key point of LR after PD.

The adhesions after laparoscopic surgery are often loose in comparison to those after laparotomy [1, 2, 9]. Therefore, we can frequently perform the second operation after laparoscopic surgery in almost the same way as the primary operation. Therefore, at the authors’ institution, the laparoscopic approach is the first choice for repeat surgery after laparoscopic interventions, and LLR was intended for the present case. However, in LLR, meticulous haemostasis is needed, and vascular control is preferable.

First, we adopted the left lateral decubitus position to identify the portal vein at the hepatic hilum and to perform partial resection of segment 5. In this position, the burden of the jejunal loop, which is located in front of the portal vein, was released to the left side of the portal vein, and we could approach the portal vein from the right side, avoiding the pancreatic fistula region. Therefore, removal of the adhesions and isolation of the portal vein were easier to perform in this position than in the supine position. Then, for left lateral sectionectomy, the patient was placed in a supine position. In this position, the portal vein was hidden beneath the hepato-jejunal anastomosis, as illustrated in Fig. 3b.

In our case, if the adhesions around the portal vein and hepatic artery were more dense than expected, we planned to resect the liver parenchyma without vascular inflow control to give up isolation of these vessels. However, the adhesion on the right side of the portal vein was somewhat loose. Therefore, we succeeded in isolating the portal vein with little sharp cuts and much blunt peeling of the adhesion. On the other hand, the adhesion around the pancreaticojejunostomy, which is close to the hepatic artery, was extremely severe. Therefore, we did not pursue complete isolation of the hepatic artery for fear of injury to this vessel. Furthermore, occlusion of the hepatic artery was only half.

As a result, the preferred procedure of LLR was performed with a blood loss of 100 g through innovative and strategic body positions, a breakthrough for isolating the portal vein. To the best of our knowledge, LLR after LPD has not been reported in the English literature, and only one report was related to LLR after open PD (but provided no details) [2]. Therefore, this may be the first successful and detailed report of this procedure.

An infectious complication of the raw surface of the liver is one of the main issues concerning hepatectomy after PD [4, 6]. Enteric bacterial colonization in the bile with biliary-enteric anastomosis is indicated, resulting in abscess formation after LR [6]. In our case, it is assumed that very little bile leakage existed on the raw surface, and it collected by undetectable degrees. This minor bile leakage of the raw surface was absorbed without clinical signs in many cases. However, in our case, the enteric bacteria from biliary-enteric anastomosis infected this bile collection. As a result, an intra-abdominal abscess on the cut surface formed, and additional drainage was needed. However, he had no signs of infection during hospitalization. This suggested that more attention should be paid to late infection after LR with biliary-enteric anastomosis compared to that after LR without biliary-enteric anastomosis.

The standard treatment for liver metastases from ampulla of Vater adenocarcinoma is chemotherapy with gemcitabine plus cisplatin [10, 11]. However, some cases of LR for liver metastases have been reported [3, 7, 8]. In these series, patients with solitary liver metastasis, a long interval from the initial operation to recurrence, and margin-negative resection had a good prognosis [3, 7, 8]. From these viewpoints, our case did not have preferable indications for LR. Therefore, at first, we believed that systemic chemotherapy was necessary, because the recurrence site was in the liver and para-aortic lymph nodes. Chemotherapy caused the disappearance of the para-aortic lymph nodes without affecting the liver metastases. Because the side effects of chemotherapy were less for him, we judged that this chemotherapy was continuable and that the para-aortic lymph nodes were in good control for the long term. Then, we removed the liver metastases to facilitate longer term survival. We planned in advance to continue the same chemotherapy to suppress the invisible surviving cancer cells of the para-aortic lymph-node metastases. The patient has been alive for 8 months, receives chemotherapy, and has not had another recurrence.

## Conclusions

Isolation of the portal vein, which is buried in the most dorsal portion of dense adhesions, is a key point for LR after PD. LLR after PD can be performed safely with an innovative body position to isolate the portal vein. If the initial operation is laparoscopic, LLR after PD can be an option.

## Data Availability

The authors declare that all the data in this article are available within the article.

## Abbreviations

CT: Computed tomography
LR: Liver resection
PD: Pancreatoduodenectomy
LLR: Laparoscopic liver resection
LPD: Laparoscopic pancreatoduodenectomy
FDG-PET: Fluorodeoxyglucose positron emission tomography
CA19-9: Carbohydrate antigen 19-9
SUV: Standardized uptake value
PV: Portal vein
HJ: Hepaticojejunostomy
